# Synergic effect and biosafety of chitosan/zinc complex nanoparticle-based carboxymethyl cellulose coatings for postharvest strawberry preservation†

**DOI:** 10.1039/d5ra00140d

**Published:** 2025-05-12

**Authors:** Ha Thi Thu Bui, Le Thi Thanh Dang, Hue Thi Nguyen, Le Thi Le, Huy Quang Tran, Thuy Thi Thu Nguyen

**Affiliations:** a Phenikaa University Nano Institute (PHENA), Phenikaa University Hanoi 12116 Vietnam thuy.nguyenthithu@phenikaa-uni.edu.vn; b Faculty of Chemistry and Environment, Thuyloi University Hanoi 11500 Vietnam; c Faculty of Biomedical Sciences, Phenikaa University Hanoi 12116 Vietnam

## Abstract

Nanotechnology represents a burgeoning field that revolutionizes various industries and sectors, including food applications. In this study, chitosan nanoparticles (CS NPs), zinc oxide nanoparticles (ZnO NPs), and chitosan–zinc complex nanoparticles (CS/Zn NPs) were prepared and incorporated into carboxymethyl cellulose (CMC) coatings to examine their strawberry preservation efficiency. CS NPs, synthesized *via* ionic gelation, appeared with a spherical shape and a relatively uniform size below 50 nm. ZnO NPs, produced through a green electrochemical method, formed larger aggregates. CS/Zn NPs were formed due to the chelation of CS NPs with Zn^2+^ present in the fresh zinc electrochemical solution. In antibacterial tests against *Escherichia coli* and *Staphylococcus aureus*, CS/Zn NPs exhibited significantly lower MIC and MBC values compared to CS NPs and ZnO NPs individually, indicating a synergistic antibacterial effect between the components. CMC coatings containing these nanoparticles were applied to strawberry surfaces and the fruits were stored at room temperature (25 °C) and in a refrigerator (5 °C). The CS/Zn-CMC coating demonstrated the most pronounced effect in preventing weight loss and decrease of titratable acidity (TA) and ascorbic acid (AA) content over storage. It effectively preserved the original appearance of strawberries, delaying browning until day 15, while the control and other coated samples were completely spoiled within this period. The cytotoxicity assessment indicated the safety of CS/ZnO-CMC coating, suggesting its potential application in fruit preservation.

## Abstract 2

CS/Zn NPs were formed by chelating CS NPs with Zn^2+^ in the fresh zinc electrochemical solution. CS/Zn NP-based CMC coating reduced the strawberry spoilage and maintained fruit quality, indicating its potential application in fruit preservation.

## Graphical abstract

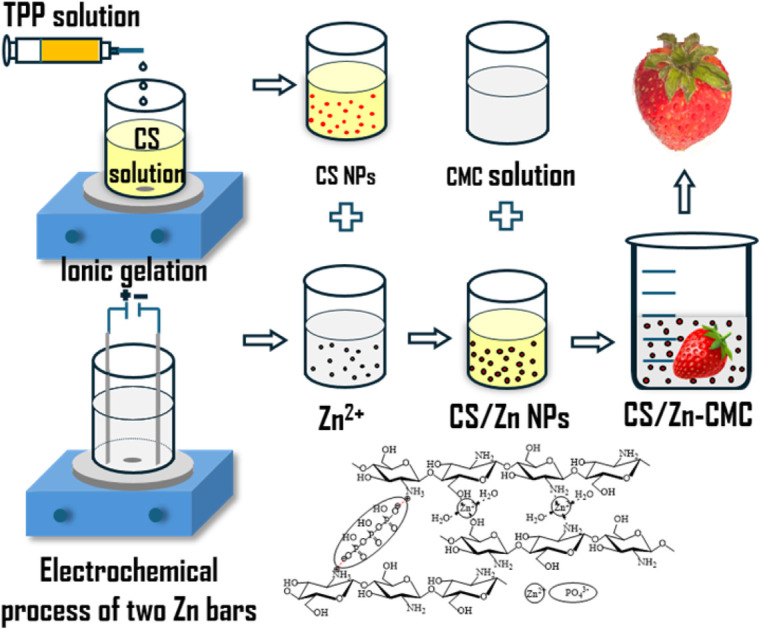

## 1 Introduction

Strawberries are a highly favorite fruit because of their attractive scent, delicious flavor, and beneficial constituents, including vitamins, anthocyanins, flavonoids, polyphenols, *etc.* (Yang *et al.*, 2022). However, their highly perishable nature makes them susceptible to physical damage, chemical changes, and biological processes during production, transportation, and storage. This results in a short shelf-life, primarily due to water loss, a high respiration rate, and microbial deterioration, causing an estimated 30% economic loss to producers and distributors.^1^ The shelf life of fresh strawberries is typically only 1–2 days at ambient temperature and prolonged to 3–5 days with cold storage at 0–4 °C.^2^

Recently, the application of coatings has been shown to be a more advanced approach to improving the quality and extending the shelf life of strawberries and other perishable fruits.^3–5^ The coatings are a thin layer of material serving as protective barriers that modify the internal atmosphere, prevent water loss, lower respiration rate, and retard microbial proliferation, which are the primary causes of fruit decay. In particular, incorporating nanomaterials into these coatings is a promising avenue for developing active coatings that effectively combat microbial spoilage. Nanomaterials, with sizes range of 1–100 nm, can notably improve the mechanical, antibacterial, and barrier properties of polymer coatings, resulting in an extension of fruit shelf life compared to coatings without nanoparticles.^6^

CS nanoparticles (CS NPs) exhibit pronounced antioxidant and antibacterial activities due to their reduced particle size and increased contact surface area. Salha *et al.*^7^ demonstrated the effectiveness of a one-step ultrasonic coating of CS NPs on strawberries, which exhibited superior inhibition of bacterial and mold growth compared to a conventional CS coating over 15 days of storage. Besides, the CS NPs coating reduced mass loss, maintained the ascorbic acid content, and delayed changes in titratable acidity and color of strawberries, suggesting great potential in strawberries preservation. However, the requirement for sonication limits its practical large-scale application. CS NPs have been combined with other compounds for the improvement of the storage quality of fruits.^8–10^ For instance, CS NPs crosslinked with guava leaf extract showed significantly improved antimicrobial properties compared to bulk CS. Therefore, the CS NPs-based coating could prevent fungal decay in strawberries to 11%, compared to 50% decay in the control, even with a low concentration of CS NPs.^9^ Another study revealed that a combination of 3% CaCl_2_ and CS NPs could reduce weight loss, preserve the content of bioactive components such as ascorbic acid and total anthocyanin, antioxidant activity, and slow down the production of malondialdehyde in strawberries stored at 4 °C up to 15 days.^10^

Among inorganic nanoparticles, zinc oxide nanoparticles (ZnO NPs) have gained considerable interest in fruit coating because of their antimicrobial broad-spectrum activities, excellent barrier capacities, biocompatibility, and food safety approved by the US Food and Drug Administration. According to Sogvar *et al.*,^11^ the treatment of strawberries with ZnO NPs decreased the microbial load during fruit storage as well as maintained fruit quality in terms of weight loss, firmness, antioxidant activity, and bioactive components. ZnO NPs have been widely incorporated into polysaccharides, lipids, and protein-based biopolymers to effectively enhance the mechanical, barrier, and physicochemical properties of fruit coatings.^6^ The incorporation of ZnO NPs into the carboxymethyl cellulose (CMC) coatings resulted in a significant decrease of water vapor permeability and oxygen transfer rate, coupled with a significant enhancement of antioxidant and antimicrobial activities against several human and plant pathogens, suggesting their potential as effective protective coatings of food products.^12^ Especially, ZnO NPs loaded CS coatings provided many benefits in the preservation of various kinds of fruits, for instance, guavas,^13^ fresh-cut papaya,^14^ passion fruit,^15^ and strawberry.^1^ A synergistic antimicrobial effect between CS and ZnO NPs was observed in CS/ZnO NPs coating, resulting in a significant reduction in aerobic mesophilic bacteria, mold, and yeast loads of strawberries stored for 8 days.^1^ Moreover, the coated strawberries retained desirable quality parameters, including pH, total soluble solids, and titratable acidity, ensuring their commercial viability. Sani *et al.*^16^ developed CS-based coatings containing ZnO NPs, lemon juice, ginger extract, and garlic extract for strawberry preservation. These coatings reduced weight loss and decay rate by approximately 40% and extended the strawberry shelf-life to 30 days when stored at 4 °C. These outcomes are attributed to the synergistic effect of CS and ZnO NPs, further enhanced by the presence of antibiotic compounds in ginger and garlic extracts. In another study, CS/ZnO NPs coatings supplemented with essential oil (EOs) prolonged the quality of table grapes, with respect to weight loss, browning, microbial contamination, and nutrient contents, for more than 4 days compared to untreated samples.^17^

CMC has been widely utilized as a coating material for fruit preservation owing to its excellent barrier and mechanical properties.^18^ CMC coatings applied to banana, mandarin, mango, and tomatoes have demonstrated positive effects on fruit quality, including prevention of weight loss, enhancement of firmness, and reduction of respiration rate and ripening index.^19^ Given the modest magnitude of these effects, further research is necessary to enhance the fruit preservation efficacy of CMC coatings. This study aims to investigate the effectiveness of CMC coatings incorporating CS NPs, ZnO NPs, and CS/Zn complex NPs in preserving strawberries. CS NPs were prepared by the ionic gelation method, which is based on electrostatic interactions to create cross-linking between positively charged chitosan and a negatively charged tripolyphosphate. ZnO NPs were produced by an electrochemical process that avoids the use of harmful chemicals. The combination of CS NPs solution and electrochemical solution formed CS/Zn complex NPs, designed to exhibit the synergic antibacterial activity of both components. Strawberries coated with these different nanoparticles-loaded CMC coatings were evaluated for weight loss, titratable acidity, ascorbic acid, decay incidence, and sensory evaluation. The findings of this research may offer a promising approach for extending the postharvest shelf life of strawberries.

## 2 Materials and methods

### 2.1 Materials

Chitosan (average MW of 30 000 Da, degree of deacetylation ≥ 95%) was purchased from Cool Chemical Science and Technology, China. Tripolyphosphate (TPP, purity ≥ 98%) and trisodium citrate (Na_3_C_6_H_5_O_7_, purity > 99%) were supplied by Sigma-Aldrich. Acetic acid (purity ≥ 99.5%) and ascorbic acid was obtained by Xilong Scientific Co. Ltd, China. Carboxymethyl cellulose sodium (CMC, extra pure) was provided by Samchun Pure Chemical Co. Ltd, Korea. Two zinc bars (99.999% purity) with length, width, and thickness dimensions of 150 × 10 × 0.5 mm, respectively, were used as electrodes for the electrochemical process. Bi-distilled water through a Milli-Q® system was used. All chemicals were used without further purification.

Two bacterial strains, namely, Methicillin-resistant *Staphylococcus aureus* (MRSA, Gram-positive bacteria) and *Escherichia coli* (*E. coli*, Gram-negative bacteria) were provided by the Department of Bacteriology, National Institute of Hygiene and Epidemiology, Vietnam.

Strawberries originating from Moc Chau, a mountain religion in the North of Vietnam, were transported to the laboratory after a one-day harvest. They were selected according to uniform size and color without visible mechanical damage and disease symptoms for each experimental group.

### 2.2 Preparation of nanoparticles

Chitosan nanoparticles (CS NPs) were prepared by an ionic gelation method which is based on electrostatic interactions to create cross-linking between positively charged chitosan (CS) and negatively charged tripolyphosphate. Briefly, 0.24 g CS was dissolved in 40 mL of 1% v/v acetic acid using a magnetic stirrer at room temperature for 12 h to obtain a homogeneous solution. Then, a quantity of Tween 80 calculated as 0.15% v/v of CS solution was added. Subsequently, 40 mL of 0.3 w/v TPP solution was introduced dropwise into the CS solution prepared above at a rate of 30 mL h^−1^ under the stirring condition at 1000 rpm. CS NPs were allowed to settle overnight and then the supernatant was decanted. 100 mL of distilled water was added, and the decantation process was repeated to obtain 30 mL of an 8.0 mg mL^−1^ CS NPs solution in the sediment.

Zinc oxide nanoparticles (ZnO NPs) were prepared by an electrochemical method using two pure zinc bars as electrodes. Briefly, two zinc bars were immersed in 200 mL of 0.02 wt% Na_3_C_6_H_5_O_7_ solution contained in a 250 mL glass beaker. The distance between the two electrodes was 5 cm and a 9 V DC voltage source was applied for 2.5 hours at room temperature. The solution was continuously stirred during the electrochemical process and then immediately used for preparing CS/Zn complex nanoparticles (CS/Zn NPs). The concentration of Zn moiety in the solution after the electrochemical process was 33 mg L^−1^ according to atomic absorption spectroscopy analysis.

To prepare a solution of CS/Zn complex nanoparticles, the fresh electrochemical solution was added to the CS NPs solution with the same volume and then stirred for 2 hours.

### 2.3 Preparation of coating layer on strawberries

CMC was used as a coating formation agent, incorporating three different types of nanoparticles, namely CS NPs, ZnO NPs, and CS/Zn NPs. Briefly, CMC was dissolved in distilled water to form a homogeneous CMC solution with a concentration of 1.25 wt%. Coating solutions of CMC, CS NPs-CMC, and ZnO NPs-CMC were prepared by mixing 20 g of a 1.25 wt% CMC solution with either 30 mL of distilled water, CS NPs solution, or ZnO NPs solution, respectively. To prepare the CS/Zn-CMC solution, 20 g of 1.25 wt% CMC solution was mixed with 15 mL of CS NPs and 15 mL of ZnO NPs solutions. Finally, the volume of each of these solutions was adjusted to 50 mL by adding distilled water. Table 1 provides the components and their concentration in each coating solution.

**Table 1:** Concentration of each component in coating solutions

Solution	CMC (g mL^−1^)	CS NPs (mg mL^−1^)	ZnO NPs (μg mL^−1^)
CMC	0.5	—	—
CS NPs-CMC	0.5	2.4	—
ZnO NPs-CMC	0.5	—	20
CS/Zn-CMC	0.5	1.2	10

Strawberries with the same size and free damage were cleaned with distilled water and divided into five groups randomly. One group was considered as control without any coatings. The remaining four groups were dipped into CMC, CS NPs-CMC, ZnO NPs-CMC, and CS/Zn-CMC solutions for 1 minute at room temperature. Strawberries in five groups were preserved at two different conditions: room temperature (25 °C and RH 75%) and refrigerator temperature (5 °C and RH 60%).

### 2.4 Characterizations of the nanoparticles

The morphology of nanoparticles (CS NPs, ZnO NPs, and CS-Zn NPs) and CMC coating containing CS-Zn NPs were observed by field emission scanning electron microscopy (FE-SEM, JSM-IT800/JEOL).

The chemical bonding in CS NPs and CS-Zn NPs was analyzed by using Fourier-transform infrared spectroscopy (FTIR, Nicolet NEXUS 670 spectrometer) in transmission mode in the wavelength range of 500–4000 cm^−1^.

X-ray diffraction (XRD) patterns of three nanoparticles were measured with Cu K_α_ radiation in a 2*θ* range from 5 to 80° using EQUINOX 5000 – Thermo Scientific X-ray diffractometer.

### 2.5 Evaluation of fruit quality

#### 2.5.1 Measurement of weight loss

The weight loss of strawberries was measured by the difference between the initial weight and the weight on 3 day intervals using the following formula:
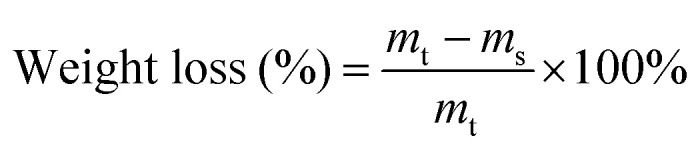
where *m*_t_ is the initial weight of strawberries and *m*_s_ is the weight of strawberries after storage intervals.

#### 2.5.2 Determination of decay incidence

Rot symptoms were identified by visual observation. During storage, strawberries became discolored, turning darker or developing white hyphae on the fruit surface. The disease index (DI) value was used to evaluate the decay incidence. Specifically, scores of the total extent of the visibly decayed area on the fruit surface based on the following scale: 0 = no evidence of decay; 1 = 1–10% decay, 2 = 11–25% decay, 3 = 26–40% decay, 4 = 40–50% decay, and 5 = >50% decays. The DI was calculated by the following equation:
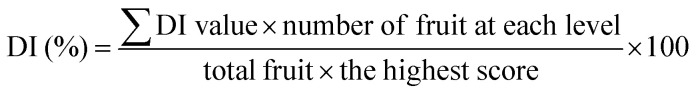


Fruits with DI scores of 2, 3, 4, or 5 are almost unmarketable.

#### 2.5.3 Measurement of titratable acidity (TA)

All strawberries in each group were determined the weight and crushed to strawberry pulp. The homogenized pulp was mixed with 100 mL of distilled water and filtered through a filter paper (15–20 μm) to obtain an extracted solution. Subsequently, two drops of 1% (w/v) phenolphthalein were added to 20 mL of the strawberry extract and the mixture was titrated with 0.1 mol L^−1^ NaOH solution when a permanent pink appeared. TA (%) value was determined by the following equation:
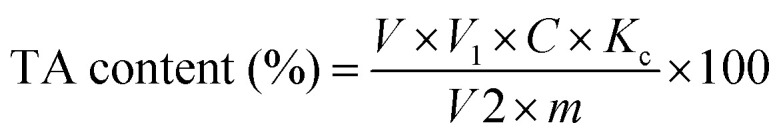
where *V* is the volume of NaOH solution consumed in titration (mL); *V*_1_ is the total volume of strawberry extract (mL); *C* is the molar concentration of NaOH solution (mol L^−1^); *K*_c_ is the milliequivalent factor of citric acid, 0.064 g mmol; *V*_2_ is the volume of strawberry extract used for titration (mL); *m* is the total mass of strawberry used to titrate (g).

This process was triplicated at each time interval until the end of storage.

#### 2.5.4 Measurement of ascorbic acid (AA) content

The KIO_3_ titration method was used to measure AA content in strawberries. All strawberries in each group were crushed to strawberry pulp. Two grams of strawberry pulp were diluted to a volume of 20 mL by 2% (v/v) hydrochloric acid solution and filtered through a filter paper (15–20 μm) to obtain an extracted solution. Then, 5 mL of strawberry extract was taken and mixed with 0.5 mL of 10 g L^−1^ KI solution. 2.0 mL of 5 g L^−1^ starch solution, and 2.5 mL of distilled water. The mixture was titrated using 1 mmol L^−1^ KIO_3_ solution until a permanent light blue appeared. AA content was determined by the following equation:
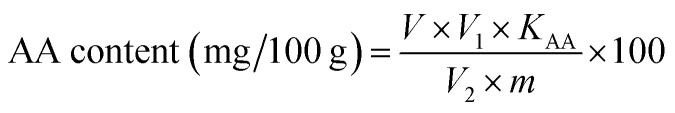
where *V* is the volume of KIO_3_ solution consumed in titration (mL); *V*_1_ is the total volume of strawberry extract; *K*_AA_ is the mass of AA equal to 1 mL of 1 mmol L^−1^ KIO_3_ solution, 0.088 mg; *V*_2_ is the volume of extract used for titration (mL); *m* is the weight of the strawberry pulp used for titration (g).

### 2.6 Antimicrobial activity of nanoparticles

The antimicrobial activity was tested against two foodborne bacteria,^20^ namely *Escherichia coli* (*E. coli*) and *Staphylococcus aureus* (MRSA) at a concentration of 5 × 10^5^ colony-forming units (CFU) per mL.

**Fig. 1:**
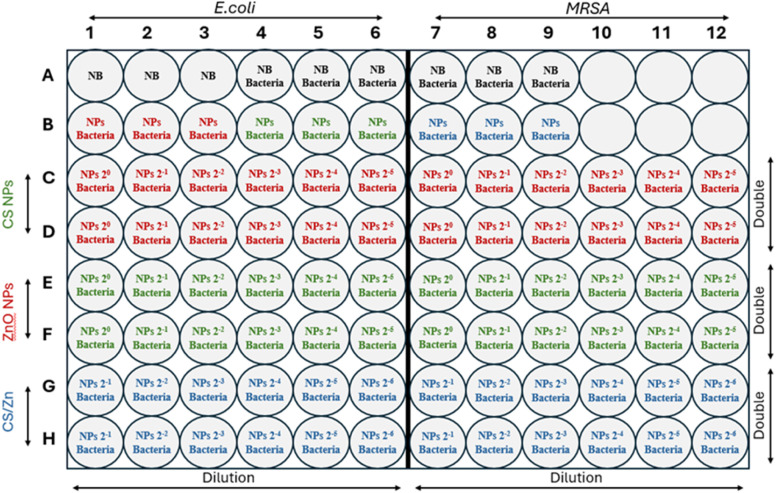
The experimental designs for antimicrobial tests of CS NPs, ZnO NPs, and CS/Zn NPs at two-fold serial concentrations against *E. coli* and MRSA.

The antimicrobial test of three nanoparticles at six different concentrations (two-fold serial concentrations) was designed in a sterilized 96-well plate (Fig. 1) as follows: row A served as negative controls, with three wells containing only nutrient broth. The next wells acted as positive controls, including three wells inoculated with *E. coli* in nutrient broth and three wells inoculated MRSA in nutrient broth. Row B contained three wells filled with each nanoparticle solution to check for bacteria contamination. In row C, twelve wells received 100 μL of CS NPs at varying concentrations and 100 μL of bacteria (*E. coli* in C1–C6 and MRSA in C7–C12). The samples in row C were repeated in row D for double measurements. Similarly, rows E and F were used for ZnO NPs and rows G and H were used for CS/Zn complex NPs. Subsequently, the plate was incubated at 37 °C for 24 h.

The antimicrobial effectiveness of nanoparticles was evaluated through minimum inhibitory concentration (MIC: the lowest concentration of nanoparticles that inhibits the visible growth of bacteria) and minimum bactericidal concentration (MBC: the lowest concentration of nanoparticles that prevents the growth of bacteria). In addition, the bacterial viability was determined by counting the bacterial colonies growing on the agar surface. Briefly, serial dilutions of nanoparticle-treated bacterial solutions from the 96-well plate were prepared using the physiological saline. 100 μL of each dilution were spread onto agar plates and incubated at 37 °C for 24 hours. The number of bacteria colonies was then enumerated to calculate the viable bacterial concentrations in the initial inoculated solutions.

### 2.7 Antioxidant activity of nanoparticles

The antioxidant properties of nanoparticles were determined using DPPH assay, which measure the free radical scavenging activity. The measurements were conducted according to the Chen *et al.* method^21^ with minor modifications. A 0.1 mM DPPH solution was prepared in methanol to form a homogeneous solution. Afterward, 2 mL of this solution was added to 2 mL of sample solution and 2 mL of distilled water as a negative control. Additionally, 2 mL of the DPPH solution was added to 2 mL of a 17 μg mL^−1^ ascorbic acid (AA) solution as a positive control. After incubating the mixtures in the dark for 30 minutes at room temperature, the absorbance of each solution was measured at 517 nm using a UV-Vis spectrophotometer. All sample was measured in triplicate, and the average result was reported. The antioxidant activity was expressed as the percentage of DPPH scavenging capacity, calculated using the following formula:
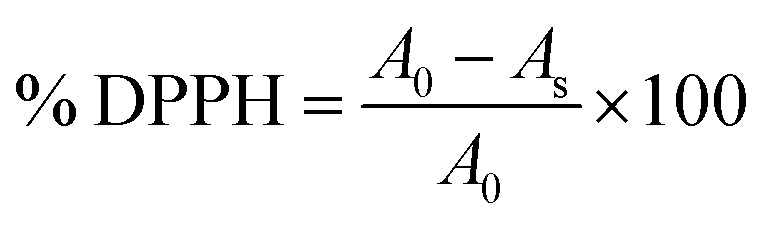
where *A*_0_ is the absorbance of control and *A*_s_ is the absorbance of sample.

### 2.8 Cytotoxicity assay

HEK293 cells were cultured at 37 °C, 5% CO_2_ in DMEM (Dulbecco's modified eagle medium) supplemented with 2 mM l-glutamine, antibiotics (penicillin and streptomycin sulfate) and 5–10% fetal bovine serum. The cell suspension was seeded onto 96-well plates (1.5 × 10^5^ cells per well) and incubated with CS/Zn NPs at concentrations ranging from 8–128 μg mL^−1^, with each concentration tested in triplicate. Dimethyl sulfoxide (DMSO ≤ 1% v/v) was used as a negative control. The optical density of the formazan metabolite in DMSO was measured at wavelength of 540/720 nm on a Tecan Spark® instrument (Switzerland). The cell proliferation inhibition ability of CS/Zn NPs was calculated as a percentage compared to the control according to the following formula:
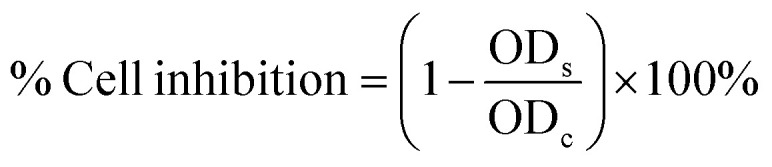
where OD_s_ and OD_c_ are the optical density of cell suspension treated with CS/Zn NPs and negative control, respectively. The sample that showed activity (% cell inhibition > 50%) were determined by IC_50_ value using TableCurve AISN software.

### 2.9 Statistical analysis

Data were statistically analyzed using one-way analysis of variance (ANOVA). Sources of variation were storage day and groups of treatment (control, CMC, CS NPs, ZnO NPs, and CS/Zn NPs samples). The data were expressed as mean ± standard error (*n* = 3). A *p*-value of less than 0.05 was considered statistically significant.

## 3 Results and discussion

### 3.1 Characterization of the nanoparticles

**Fig. 2:**
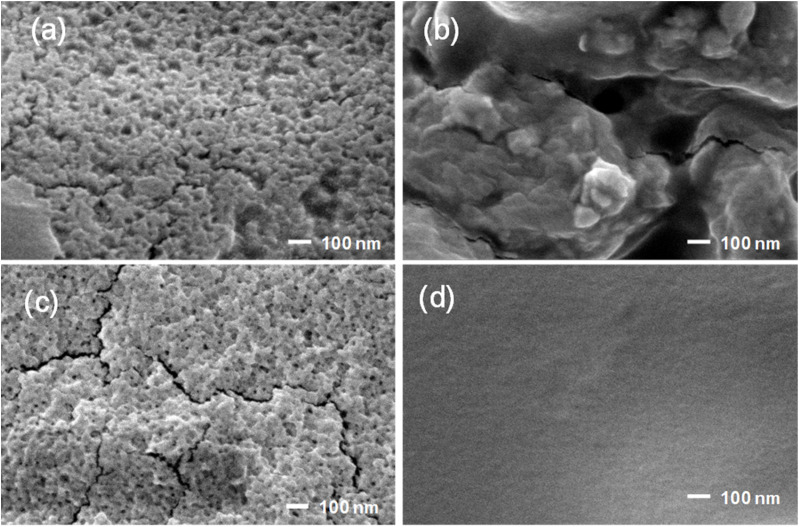
SEM images of CS NPs (a), ZnO NPs (b), CS-Zn NPs (c), and CMC coating containing CS/Zn NPs (d).

**Fig. 3:**
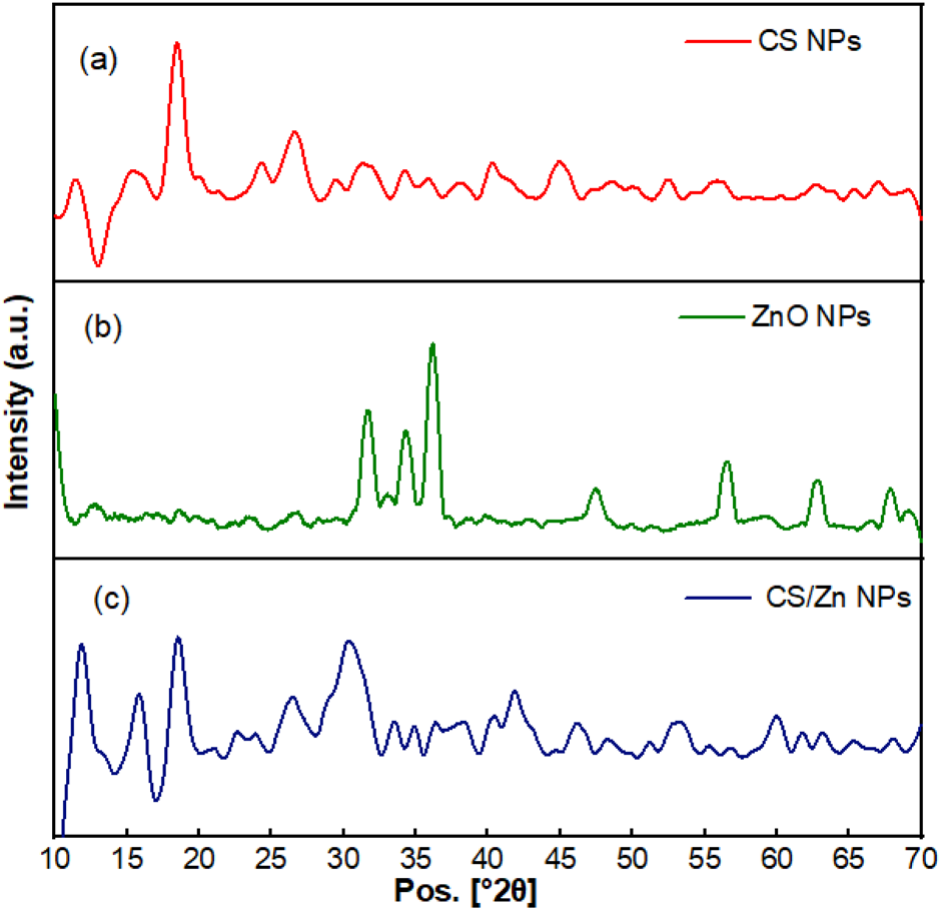
XRD pattern of CS NPs (a), ZnO NPs (b), and CS/Zn NPs (c).

Fig. 2 displays SEM images of prepared CS NPs, ZnO NPs, and CS/Zn NPs and CMC coating containing CS/Zn NPs. It can be observed that the synthesized CS NPs exhibited a spherical morphology with relatively uniform sizes of less than 50 nm (Fig. 2a). ZnO NPs appeared with uneven size, considerably larger than CS NPs (Fig. 2b). The electrochemical dissolution of zinc anode in an aqueous electrolyte produced Zn^2+^ ions in solution. These ions subsequently reacted with hydroxide ions (OH^−^) released from the cathode to produce Zn(OH)_2_ which further dehydrate to form ZnO nuclei.^22^ Over time, the ZnO nuclei underwent aggregation and growth, resulting in the formation of large ZnO NPs. As a result, the solution from an electrochemical process contained a mixture of residual Zn^2+^ and ZnO NPs of varying sizes. However, when the newly formed electrochemical solution of Zn bars was mixed with the solution of CS NPs, the obtained nanoparticles had a similar morphology with CS NPs and individual ZnO NPs were not distinguished on the SEM image (Fig. 2c). The hypothesis was proposed that active sites of chitosan molecules chelated Zn^2+^ ions or ZnO nuclei by donating electron pairs from oxygen atoms in hydroxide groups or nitrogen atoms in amino groups. In other words, chitosan hindered the growth of ZnO nuclei in the electrochemical solution and chitosan–zinc complex nanoparticles (CS–Zn NPs) were formed. The surface morphology of the CMC coating containing CS/Zn NPs (Fig. 3d) was characterized by a smooth surface and uniform appearance with no discernible particle boundaries or defects, suggesting excellent miscibility between CS/Zn NPs and the CMC matrix.

The X-ray diffraction (XRD) pattern of CS NPs, ZnO NPs and CS/Zn NPs were analyzed to confirm the phase formation of nanoparticles (Fig. 3). The XRD pattern of ZnO NPs showed diffraction peaks at scattering angles (2*θ*) of 31.8°, 34.5°, 36.1°, 47.4°, 56.6°, 62.9°, and 67.9° corresponding to the 100, 002, 101, 102, 110, 103, and 112 planes, respectively, of hexagonal structure of ZnO NPs (JCPDS card no. 36-1451). The characteristic peaks of CS NPs were observed at 2*θ* of 11.5°, 18.5°, 24.5°, 26.8°, 32.0°, 34.2°, 40.0°, and 45°. The high intensity of diffraction peak at 18.5° demonstrated that the CS NPs possessed a high degree of crystallinity.^23–25^ Ahmed *et al.*^26^ proposed that the ionic crosslinking between the amino groups of CS and the phosphate groups of TPP could lead to a more crystalline order for CS NPs. When the CS NPs were mixed with a fresh electrochemical solution of Zn bars, the characteristic peaks of CS NPs exhibited alterations in intensity and scattering angles, suggesting an interaction between CS NPs and Zn^2+^ ions or ZnO nuclei, resulting in the formation of CS/Zn complex NPs. Notably, the XRD pattern of CS/Zn NPs revealed the complete disappearance of diffraction peaks associated with ZnO NPs. Perelshtein *et al.*^27^ reported a similar result when synthesizing CS/Zn complex *via* the sonochemical reaction of dissolved CS and zinc acetate solution at pH 8. They proposed that the Zn/CS complex consisted of ZnO particles with a size of a few nanometers, potentially too disordered or small to be detectable by XRD. Employing additional analytical techniques, they further confirmed the existence of nanocrystalline ZnO with an average particle size of less than 2 nm within the Zn/CS complex.

**Fig. 4:**
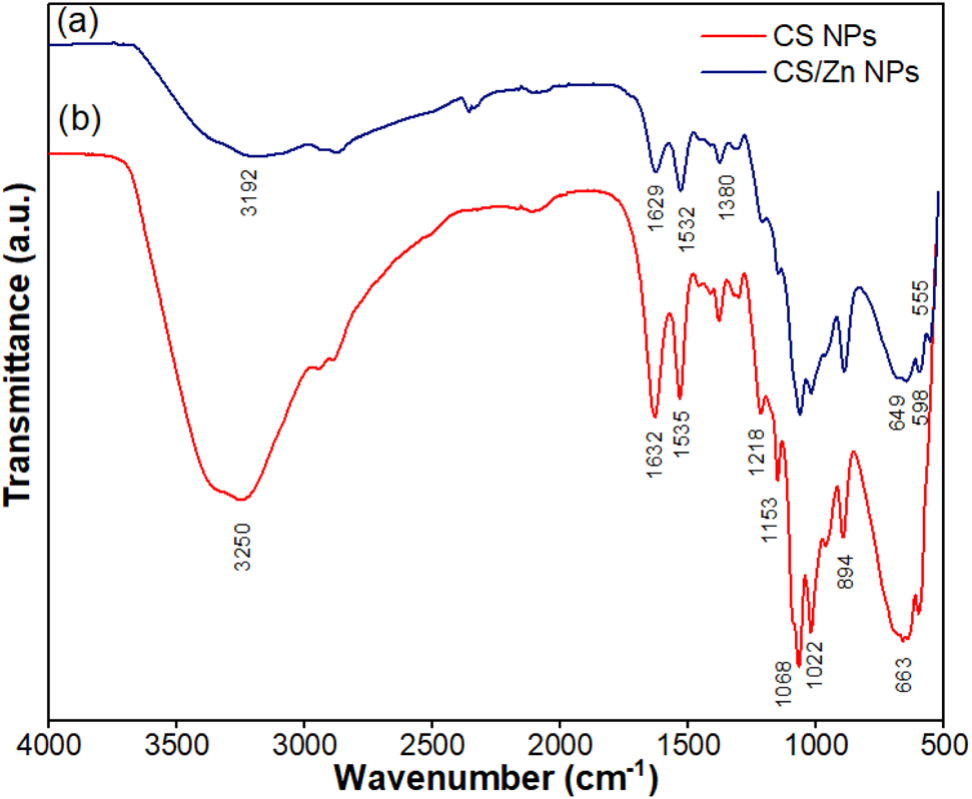
IR spectra of CS NPs (a) and CS/Zn NPs (b).

To further confirm the interaction found in the CS/Zn complex NPs, IR analysis of CS NPs and CS/Zn NPs was performed (Fig. 4). The IR spectrum of CS NPs showed a strong peak at 3250 cm^−1^ representing the overlapped stretching vibrations of –OH and –NH_2_ groups of CS. Absorption peaks at 1632 cm^−1^ and 1535 cm^−1^ were associated with –C=O stretching vibration (amide I) and –N–H stretching vibration (amide II), respectively. The presence of an absorption peak at 1380 cm^−1^ indicated the –CH_3_ wagging. Asymmetric stretching vibration of glycosidic linkage –C–O–C– appeared at 1153 cm^−1^, while –C–O stretching vibration was assigned at 1068 cm^−1^. Crosslinked CS was characterized by an absorption peak at 1218 cm^−1^ corresponding to –P=O stretching vibration.^28^ Two peaks at 1022 cm^−1^ and 894 cm^−1^ were attributed to the symmetric and asymmetric stretching vibration of the –PO_3_^2−^, respectively, due to the crosslink of protonated amino groups of CS with TPP.^28^ In comparison with CS NPs, IR spectrum of CS/Zn NPs shows that absorption peaks characterized by stretching vibrations of –OH and –NH_2_ groups of CS became broader, accompanied by the reduction of intensity of all absorption peaks of crosslinked CS NPs. Additionally, the two peaks assigned to the stretching vibration of –C=O and –N–H shifted to a lower wavelength. This evidence suggested that the –NH_2_ and –OH groups are involved in the complexation with Zn^2+^ ions in the electrochemical solution.

### 3.2 Antimicrobial activity of nanoparticles

**Fig. 5:**
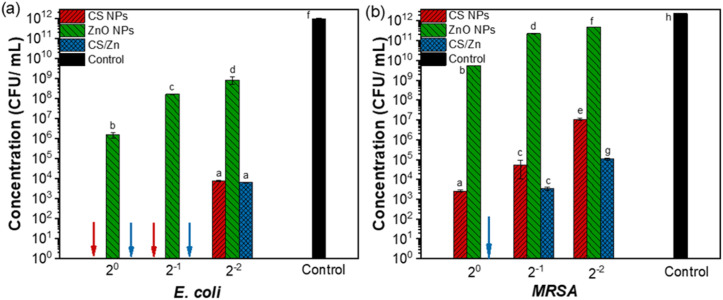
The antibacterial effectiveness of CS NPs, ZnO NPs, and CS/Zn NPs against *E. coli* (a) and MRSA (b). Different letters indicate significant statistical differences among groups, *n* = 3, *p* < 0.05.

Microbial proliferation, originating from both endogenous sources within the fruit and exogenous contamination from the external environment, is a primary cause of fruit spoilage during storage and poses a significant risk to human health. Consequently, antimicrobial properties constitute a crucial characteristic of coating materials designed for fruit preservation. This study evaluated the antimicrobial efficacy of CS NPs, ZnO NPs and CS/Zn NPs against *E. coli* (a Gram-positive bacteria) and MRSA (Gram-negative bacteria), both common food-borne pathogens. The antibacterial activity of these materials at various concentrations was quantified and visualized in Fig. 5, Table 2, and ESI data 1.† CS NPs demonstrated bactericidal activity against *E. coli* at their initial concentration and when diluted twofold. At a fourfold dilution, CS NPs exhibited an inhibitory effect, resulting in a survival rate of 7.4 × 10^3^ CFU mL^−1^. ZnO NPs displayed an inhibitory effect against *E. coli* with survival rates of 1.5 × 10^6^, 1.6 × 10^8^, and 8.25 × 10^8^ CFU mL^−1^ at initial concentration, twofold dilution, and fourfold dilution, respectively. As depicted in Fig. 5a, the antibacterial activity of CS/Zn NPs was comparable to that of CS NPs against *E. coli*, suggesting that they are primarily attributable to the CS component. All three types of nanoparticles showed lower antibacterial efficacy against MRSA than against *E. coli* at various concentrations, except for CS/Zn NPs, which completely eliminated MRSA at the initial concentration (Fig. 5b). Whereas individual CS NPs and ZnO NPs demonstrated solely inhibitory effects. At the other diluted concentration, the proliferation of MRSA treated with CS/Zn NPs was notably lower than that treated with CS NPs and ZnO NPs alone. Besides, Table 2 reveals that the MIC and MBC values of CS/Zn NPs were reduced by more than 50% compared to those of CS NPs and ZnO NPs, suggesting a synergistic antibacterial effect of components in CS/Zn NPs, consistent with previous research.^29–31^

**Table 2:** MIC and MBC values of CS NPs, ZnO NPs, and CS/Zn NPs

Samples	MIC value		MBC value	
	*E. coli*	MRSA	*E. coli*	MRSA
CS NPs (mg mL^−1^)	<0.15	1.2	2.4	>4.8
ZnO (μg mL^−1^)	5.0	20.0	>20.0	>20.0
**CS/Zn (mg mL** ^−1^ **; μg mL** ^**−1**^ **)**	<0.075 (CS) 0.31 (Zn)	1.2 (CS), 5.0 (Zn)	1.2 (CS), 5.0 (Zn)	>2.4 (CS), 10.0 (Zn)

The antimicrobial activity of CS NPs and ZnO NPs, as well as their synergistic effect in CS/Zn complexes, can be attributed to several mechanisms. Positively charged amino groups on CS NPs electrostatically interact with the negatively charged bacterial cell wall, increasing membrane permeability and leading to leakage of intracellular components. ZnO NPs release of Zn^2+^ ions, which disrupt the bacterial cell wall and interfere with DNA replication, thus exerting their antibacterial effect. The synergistic enhancement observed in CS/Zn complexes is likely due to the increased positive charge on the CS amino groups, facilitating stronger binding to the bacterial surface. This disruption of membrane integrity allows more ZnO NPs and Zn^2+^ ions to penetrate the cell, further impairing bacterial metabolic processes.^27,31^ CS NPs have demonstrated a broad spectrum of antibacterial activity with varying efficacy against Gram-negative and Gram-positive bacterial strains, potentially due to differences in cell membrane composition.^32^ While some studies suggest CS NPs are more effective against Gram-positive bacteria, others indicate greater sensitivity in Gram-negative bacteria.^33,34^ Furthermore, the antibacterial effectiveness of CS NPs is influenced by several factors such as the molecular weight and de-acetylation degree of CS, as well as environmental conditions such as pH and temperature.^32^

### 3.3 Antioxidant activity of nanoparticles

**Fig. 6:**
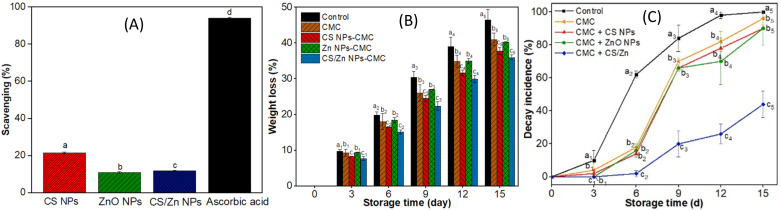
(A) DPPH free radical scavenging activity of CS NPs (2.4 mg mL^−1^), ZnO NPs (20.0 μg mL^−1^), CS/Zn NPs (2.4 mg mL^−1^, 20.0 μg mL^−1^), and ascorbic acid (17 μg mL^−1^). (B) Weight loss of strawberries during 15 days of storage. (C) Decay incidence of control and coated strawberries stored at 5 °C. Different letters indicate significant statistical differences among groups, *n* = 3, *p* < 0.05.

Oxygen availability during fruit storage is a critical factor promoting the growth of molds and anaerobic bacteria. Moreover, it can lead to the oxidative degradation of nutrients, pigments, and vitamins, resulting in undesirable changes in texture, flavor, color, and nutritive value, ultimately reducing food quality for human consumption. Applying fruit coating containing antioxidant compounds can inhibit the oxidation of fruit constituents through the oxygen-scavenging mechanism, thereby extending its shelf life. This study investigates the antioxidant activity of nanoparticles to assess their potential role in fruit preservation. Fig. 6A presents the DPPH free radical scavenging activity of CS NPs (2.4 mg mL^−1^), ZnO NPs (20.0 μg mL^−1^), CS/Zn NPs (2.4 mg mL^−1^, 20.0 μg mL^−1^), and ascorbic acid (17.0 μg mL^−1^). CS NPs exhibited a low scavenging activity of 14%, consistent with previous reports.^35,36^ The antioxidant activity of CS is attributed to its hydroxyl and amino groups, which can donate hydrogen or share lone pairs of electrons to neutralize free radicals. The reduced antioxidant capacity of CS NPs can be explained by the consumption of amino groups during the crosslinking of CS with TPP. In this study, the free radical scavenging activity of ZnO NPs, prepared by electrochemical method, was measured as 7.9% at a concentration of 20.0 μg mL^−1^. Previous studies indicate that the antioxidant activity of ZnO NPs varied by its concentration and synthesis method; for instance, scavenging activities of 40.58% (125 μg mL^−1^) have been reported for ZnO NPs synthesized using *Polygala tenuifolia* root extract,^37^ 54.3% (100 μg mL^−1^) for those synthesized from *Aerva persica* root extract,^38^ and 48.78% (125 μg mL^−1^) for ZnO NPs synthesized from *Scutellaria baicalensis* root.^39^ Apparently, the antioxidant activity of CS/Zn NPs was found to be lower than that of CS NPs, likely due to the chelation of Zn with hydroxyl and amino groups in CS.

### 3.4 Preservation quality of CS/Zn-CMC coatings on strawberry

#### 3.4.1 Weight loss

Weight loss during fruit storage is primarily attributed to respiration and moisture evaporation, leading to fruit wrinkling and deterioration. Consequently, monitoring weight loss is a critical method for assessing the efficacy of coating in the preservation of strawberries. Fig. 6B illustrates the weight loss of strawberries coated with CMC and CMC containing different nanoparticles over a 15 day storage period at a refrigerator temperature of 5 °C. While both uncoated and coated strawberries exhibited increased weight loss over time, a significant difference (*p* ≤ 0.05) was observed between the groups, particularly pronounced after 6 days. Specifically, control strawberries exhibited weight losses of 19.9% on day 6 and 46.5% on day 15, whereas strawberries coated with CMC had weight losses of 18.0% and 41.0% on the same days, respectively. Previous studies have indicated that CMC coating prevent weight loss in strawberries.^2,40,41^ The addition of ZnO NPs to the CMC coating did not result in any further decrease in weight loss compared to CMC alone. Meanwhile, coating strawberries with CMC containing CS NPs resulted in lower weight losses, which were 16.5% and 37.8% on days 6 and 15, respectively. Notably, the lowest weight loss achieving with CMC containing CS/Zn NPs, resulting in weight losses of 15.2% and 36.0% on days 6 and 15, respectively. SEM images of the CS/Zn-CMC membrane revealed a uniform and continuous structure without defects, forming an effective semi-permeable barrier that mitigated water evaporation. Additionally, this coating also reduced oxygen uptake by the strawberries, thereby slowing the rate of respiration associated with the weight loss from the fruit surface.^10,40^

#### 3.4.2 TA and AA content

TA value is a critical parameter for assessing fruit flavor and significantly impacts consumer acceptance. Concurrently, AA is an important component involved in various biochemical processes that contribute to the development of aroma and flavor compounds during fruit ripening.^15^Table 3 presents the TA and AA content of uncoated and coated strawberries stored at 5 °C. Across all samples, TA values gradually decreased over the storage period. Notably, coated strawberries consistently maintained higher TA levels compared to uncoated samples over storage days. This difference in TA content between coated and uncoated samples became more significant with longer storage duration. The decrease in TA value during strawberry storage is a well-documented phenomenon, attributed to the consumption of organic acids in respiratory and metabolic processes. Various coatings have been shown to mitigate this decline in TA. For instance, ZnO NPs coating has effectively preserved TA in strawberries,^11^ and CS coating has shown benefits for maintaining TA in passion fruit and guava, both independently and in combination with ZnO NPs.^15,26^ This preservation effect is likely due to the modification of endogenous O_2_ and CO_2_ levels by these coatings, thereby inhibiting respiratory activity in the fruits.^26^

**Table 3:** TA and AA contents of strawberries stored at 25 °C for 15 days^a^

	Storage time (day)	Groups				
		Control	CMC	CS NPs-CMC	ZnO NPs-CMC	CS/Zn NPs-CMC
TA (%)	0	0.99 ± 0.03^A^	0.99 ± 0.03^A^	0.99 ± 0.03^A^	0.99 ± 0.03^A^	0.99 ± 0.03^A^
	4	0.74 ± 0.02^B^	0.79 ± 0.03^a^	0.81 ± 0.04^a^	0.79 ± 0.02^a^	0.75 ± 0.03^a^
	8	0.50 ± 0.01^C^	0.55 ± 0.01^b^	0.52 ± 0.02^b^	0.51 ± 0.02^b^	0.54 ± 0.03^b^
	12	0.24 ± 0.02^D^	0.32 ± 0.03^c^	0.29 ± 0.01^c^	0.34 ± 0.02^c^	0.35 ± 0.02^c^
	15	0.21 ± 0.02^E^	0.24 ± 0.02^d^	0.27 ± 0.02^d^	0.27 ± 0.01^d^	0.29 ± 0.02^d^
AA (mg/100 g)	0	17.28 ± 0.10^A^	17.28 ± 0.10^A^	17.28 ± 0.10^A^	17.28 ± 0.10^A^	17.28 ± 0.10^A^
	4	16.28 ± 0.19^B^	16.54 ± 0.13^Aa^	16.71 ± 0.42^Ab^	16.96 ± 0.04^Cab^	17.01 ± 0.04^C^
	8	14.31 ± 0.04^D^	14.43 ± 0.12^Dc^	15.14 ± 0.13^Dd^	15.37 ± 0.29^Ddc^	14.81 ± 0.33^E^
	12	13.73 ± 0.12^F^	13.58 ± 0.26^Ee^	13.07 ± 0.26^Ef^	13.85 ± 0.29^Ffe^	13.58 ± 0.26^H^
	15	10.79 ± 0.16 ^G^	11.26 ± 0.13^Gh^	10.97 ± 0.42 ^Gg^	11.65 ± 0.15^Ggh^	11.97 ± 0.50^M^

a Different letters in the same column and the same row indicate significant statistical differences among groups, *n* = 3, *p* < 0.05.

Similarly, AA content in strawberries across all groups gradually reduced over storage time. This loss of AA was primarily attributed to the enzymatic activity of ascorbate oxidase, which catalyzes the conversion of ascorbic acid to dehydroascorbic acid. However, coated strawberries showed higher AA levels compared to the control group. Sogvar *et al.*^11^ proposed that ZnO NPs coating might reduce oxygen diffusion, thereby reducing the respiration rate and delaying the oxidative degradation of ascorbic acid in the fruit. Furthermore, ZnO NPs coating was associated with a reduction in moisture loss, which can further slowdown the oxidation rate. Differently, Nguyen *et al.*^10^ reported an initial increase in AA levels during the first 15 days of storage, followed by a decline in the last three days using calcium chloride-containing CS NPs coating. These discrepancies observed in these findings may be influenced by various factors, including the properties of the coating materials, the conditions under which the fruit is cultivated, and the fruit maturity level at harvest.^1,42^ In this study, while the nanoparticles exhibited limited direct antioxidant activity *via* oxygen scavenging; they likely formed a physical barrier on the fruit surface, thus restricting oxygen penetration and contributing to AA preservation.

#### 3.4.3 The visual appearance of the preserved strawberries and decay incidence

**Fig. 7:**
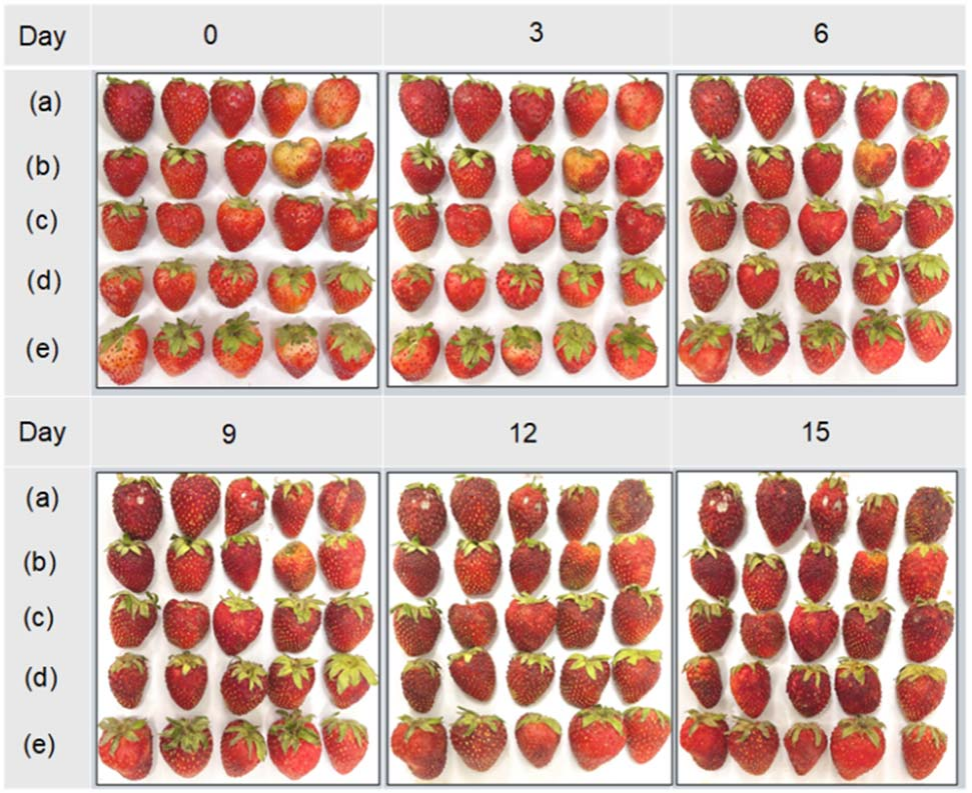
The images of five groups: control (a), CMC (b), CS NPs/CMC (c), ZnO NPs/CMC (d), CS/Zn-CMC (e) of strawberries stored at 5 °C.

Strawberries, being highly perishable, are susceptible to both mechanical damage and microbial invasion. To evaluate the efficacy of nanoparticle-based coatings, uncoated strawberries and those coated with CMC, CS NPs-CMC, ZnO NPs-CMC, and CS/Zn NPs-CMC were stored at 5 °C and 25 °C. Visual assessments of the strawberries are displayed in Fig. 7 and ESI data 2.† As shown in Fig. 7, the control strawberries began to deterioration as early as day 3, exhibiting a loss of gloss, wrinkling, and color darkening. This color change may be associated with the enzymatic oxidation of phenolic compounds, resulting in the formation of brownish pigments.^43^ Deterioration spots, indicative of microbial growth (molds, yeast, and bacteria), appeared on the surface from the third day of storage, ultimately causing fruit decay. While CMC, CS NPs-CMC, and ZnO NPs-CMC coatings could delay these deteriorative processes, CS/Zn-CMC coating was able to completely prevent fruit spoilage after day 3. Notably, the CS/Zn-CMC coating effectively maintained the original appearance of the strawberries. By day 6, CS/Zn-CMC-coated strawberries retained the commercial acceptability of fresh strawberries and maintained an appropriate acidity level (TA value in a range from 0.60 to 0.82%),^1^ indicating effective preservation. After 15 days of storage, the strawberries coated with CS/Zn-CMC appeared minimally browning, while the control and other coated groups were completely spoiled. When stored at room temperature, control strawberries deteriorated rapidly, showing significant rot on the first day (ESI data 2†). Mold growth and loss of firmness were observed in coated groups by day 2, but these effects were less pronounced with the CS/Zn-CMC coating.

The decay incidence of control and coated strawberries stored at 5 °C and 25 °C is shown in Fig. 6C and ESI data 3† showed. At 5 °C, the deterioration of control strawberries increased rapidly over storage period, reaching a 100% the decay index by day 15. While CMC, CS NPs-CMC, and ZnO NPs-CMC coatings effectively delayed spoilage, maintaining negligible decay incidence on day 3 and less than 20% on day 6, the CS/Zn-CMC coating significantly inhibited decay until day 6 and suppressed decay incidence to 44% by day 15. These findings suggest that the CS/Zn-CMC coating offers a promising approach for extending strawberry shelf life and mitigating post-harvest losses. The potent antibacterial activity of CS/Zn-CMC NPs probably contributed to this decay reduction of strawberries during storage. According to the reported studies, both CS and ZnO NPs played a role as antifungal agents, synergistically inhibiting microbial growth.^9,11^ Additionally, the CMC coating containing CS/ZnO NPs has been shown to decrease the oxygen uptake, respiration rate, water loss, and physical damage of strawberries, thereby retarding microbial growth in coated fruits.^1,43^ Similarly, Ali *et al.*^9^ reported a fungal decay percentage of around 40% in strawberries coated with CS NPs containing guava leaf extract after 12 days of storage at 4 °C and 70% RH. Thanks to the antibacterial activity of CS, essential oils (EOs), and silver nanoparticles (Ag NPs), the strawberries packed by CS–EOs–Ag NPs coatings had a decay percentage of 38% when stored at 4 °C on 12 days.^44^ In another study, ZnO NPs coating reduced strawberry waste by 20% after 18 days of storage, attributing to the preservation of fruit quality and the inhibition of microbial deterioration.^11^

### 3.5 Safety of CS/Zn-CMC coating

CS and ZnO nanoparticles are materials with widespread applications in biomedicine and food industry. Several studies have assessed the biosafety of CS and ZnO, considering factors such as size, surface area, and dosage. Using the MTT assay, Sandiz *et al.*^45^ reported no obvious cytotoxic effects of ZnO NPs within a concentration range of 1–1000 μg mL^−1^. Conversely, other studies have demonstrated the cytotoxicity of ZnO NPs in Vero cells (0.05–100 μg mL^−1^),^46^ Glial A172 cells (10–25 μg mL^−1^),^47^ and human hepatocyte L02 cell (50–100 μg mL^−1^).^48^ Similarly, CS NPs showed minimal toxicity in several normal cells, including fibroblast L929 cells (<64.21 mg mL^−1^) and NCF-12 F normal cells (10–100 μg mL^−1^) However, cytotoxicity was observed in bipotential human liver cells (0.001–1% w/v), RAW 264.7 cells (>2500 μg mL^−1^), and Zebrafish liver cells (5–10 μg mL^−1^).^49^ In this study, MTT assays revealed that CS/Zn NPS were non-toxic to the T human embryonic kidney cells (HEK293) at concentrations up to 128 μg mL^−1^. Consistent with this, other research on CS-ZnO NPs cytotoxicity in normal human A549 cells and 293T reported IC_50_ values of 20 μg mL^−1^ (ref. 50) and above 125 μg mL^−1^,^51^ respectively. Our experiment data calculated the concentration of CS/Zn NPs in the coated strawberry mass to be 5.6 mg g^−1^, corresponding to 37 μg mL^−1^, which is significantly below reported cytotoxic levels, indicating the potential safety of CS/Zn NPs for strawberry preservation.

## 4 Conclusions

This study developed and evaluated CMC coatings incorporating three distinct nanoparticles (CS NPs, ZnO NPs, and CS/Zn NPs) for their effectiveness in extending the shelf life of strawberries after harvesting. Notably, CS/Zn NPs exhibited superior antibacterial activity against *E. coli* and MRSA. The MIC and MBC values of CS/Zn NPs were substantially lower, by more than 50%, than those of CS NPs and ZnO NPs, indicating a synergistic antibacterial effect of components in CS/Zn NPs. As a result, CS/ZnO-CMC coating significantly inhibited the decay of strawberries until day 6 and suppressed decay incidence to 44% on day 15, whereas the control and other coated samples reached the decay index of almost 100%. CS/Zn-CMC coating presented a uniform and defect-free structure, forming an effective semi-permeable barrier that significantly reduced the weight loss of strawberries. After 15 days of storage, the weight loss of CS/Zn-CMC coated strawberries was 36.0%, compared to 46.5% for the control group. While TA and AA levels in strawberries gradually declined during storage, CS/ZnO-CMC-coated strawberries maintained acceptable appearance, TA, and AA levels suitable for commercial purposes until day 6. The results highligh the potential of CS/ZnO-CMC coating to minimize post-harvest losses and preserve fruit quality. Furthermore, the concentration of CS/Zn NPs in coated strawberries remained below cytotoxic levels, indicating the safety of CS/Zn-CMC coating.

## Data availability

The raw data files are available from the corresponding author upon reasonable request.

## Author contributions

The authors confirm contribution to the paper as follows: study conception and design: Thuy Thi Thu Nguyen and Le Thi Thanh Dang; data collection: Ha Thi Thu Bui, Le Thi Le, and Hue Thi Nguyen; analysis and interpretation of results: Thuy Thi Thu Nguyen and Ha Thi Thu Bui; draft manuscript preparation: Ha Thi Thu Bui; revised manuscript: Thuy Thi Thu Nguyen and Huy Quang Tran. The authors read and approved the final manuscript.

## Conflicts of interest

The authors have no competing interests or personal relationships that could have appeared to influence the work reported in this paper.

## Supplementary Material

RA-015-D5RA00140D-s001
