# Supplementary material for: Synergic effect and biosafety of chitosan/zinc complex nanoparticle-based carboxymethyl cellulose coatings for postharvest strawberry preservation
Source: RSC Adv. 2025 May 12;15(20):15539–49. doi: 10.1039/d5ra00140d (PMC12067194; doi:10.1039/d5ra00140d)
Supplement: RA-015-D5RA00140D-s001 [file RA-015-D5RA00140D-s001.pdf]

This journal is © The Royal Society of Chemistry 2025

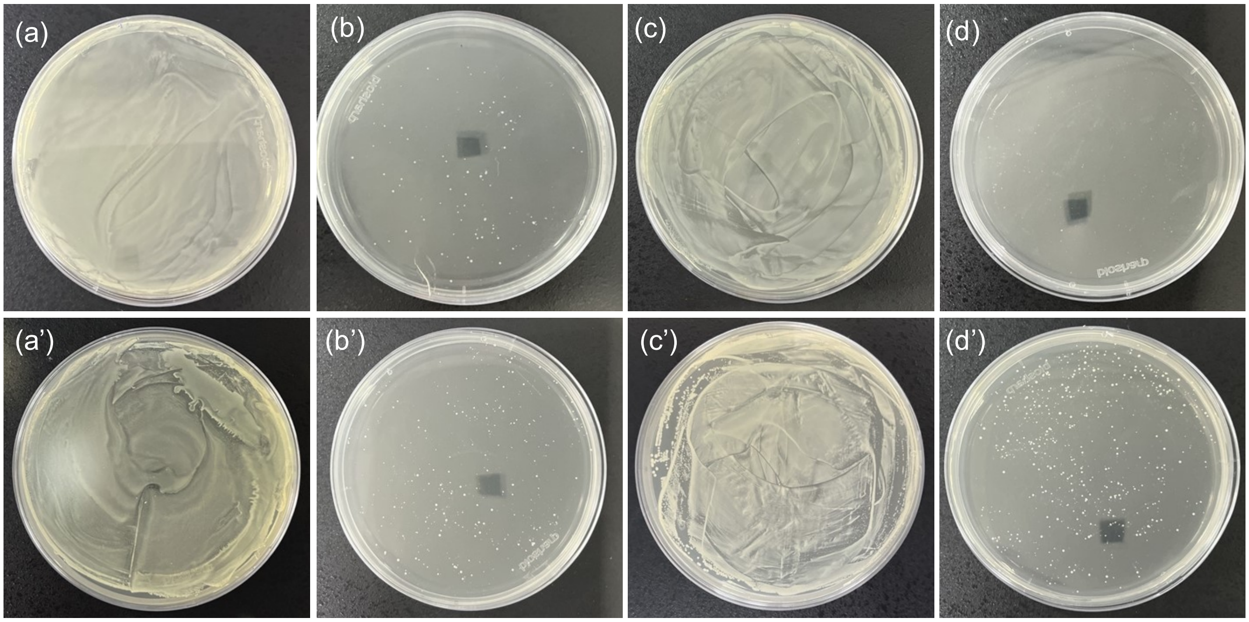

Supporting data 1. Agar plates inoculated with *E. Coli* (a, b, c, d) and MRSA (a’, b’, c’, d’), which were untreated (a, a’) and treated with CS NPs (b, b’), ZnO NPs (c, c’), and CS/Zn NPs (d, d’) with four-fold diluted concentration

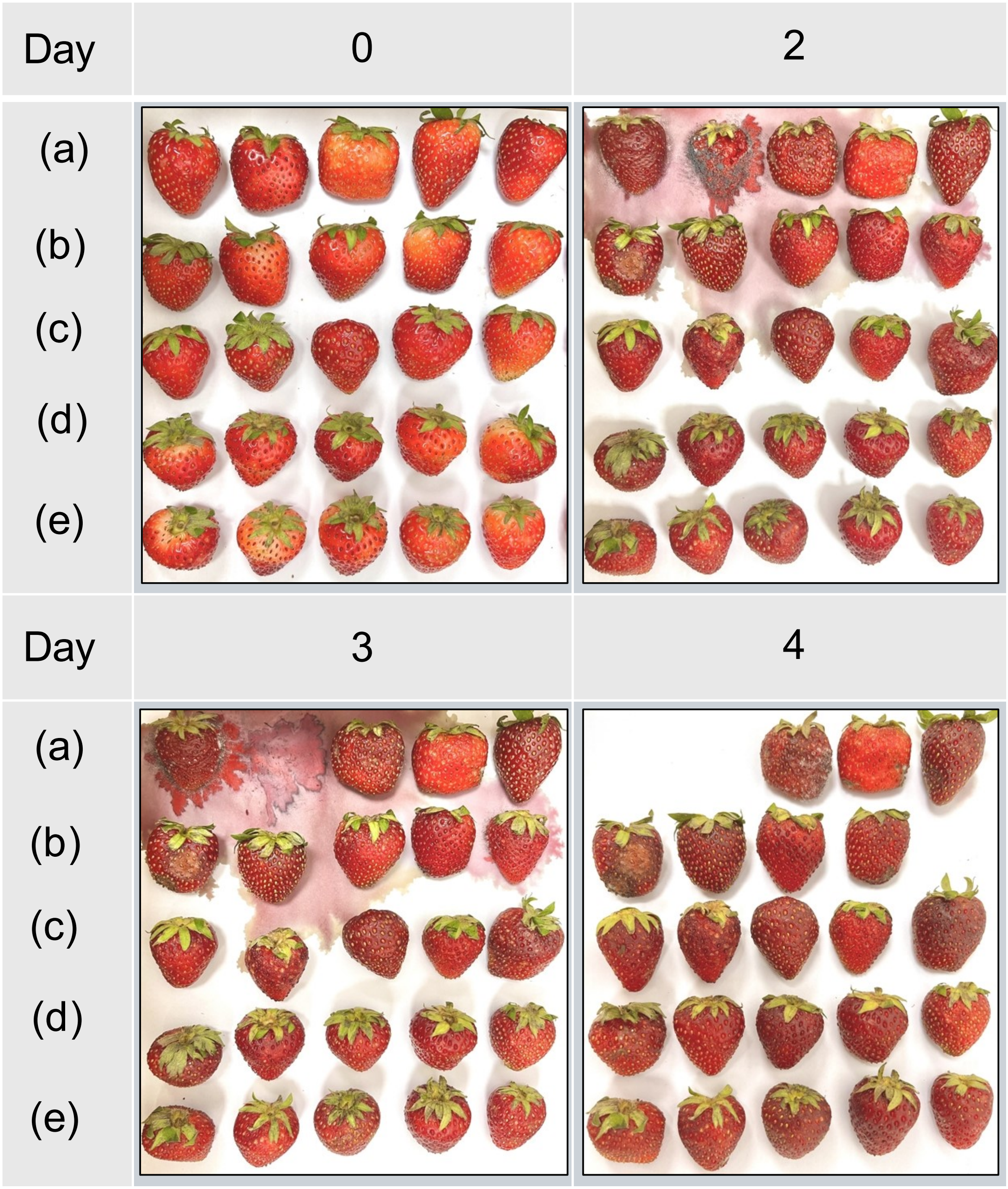

Supporting data 2. The images of five groups: control (a), CMC (b), CS NPs-CMC (c), ZnO NPs-CMC (d), CS/Zn-CMC (e) of strawberries stored at 25 ^{o}C

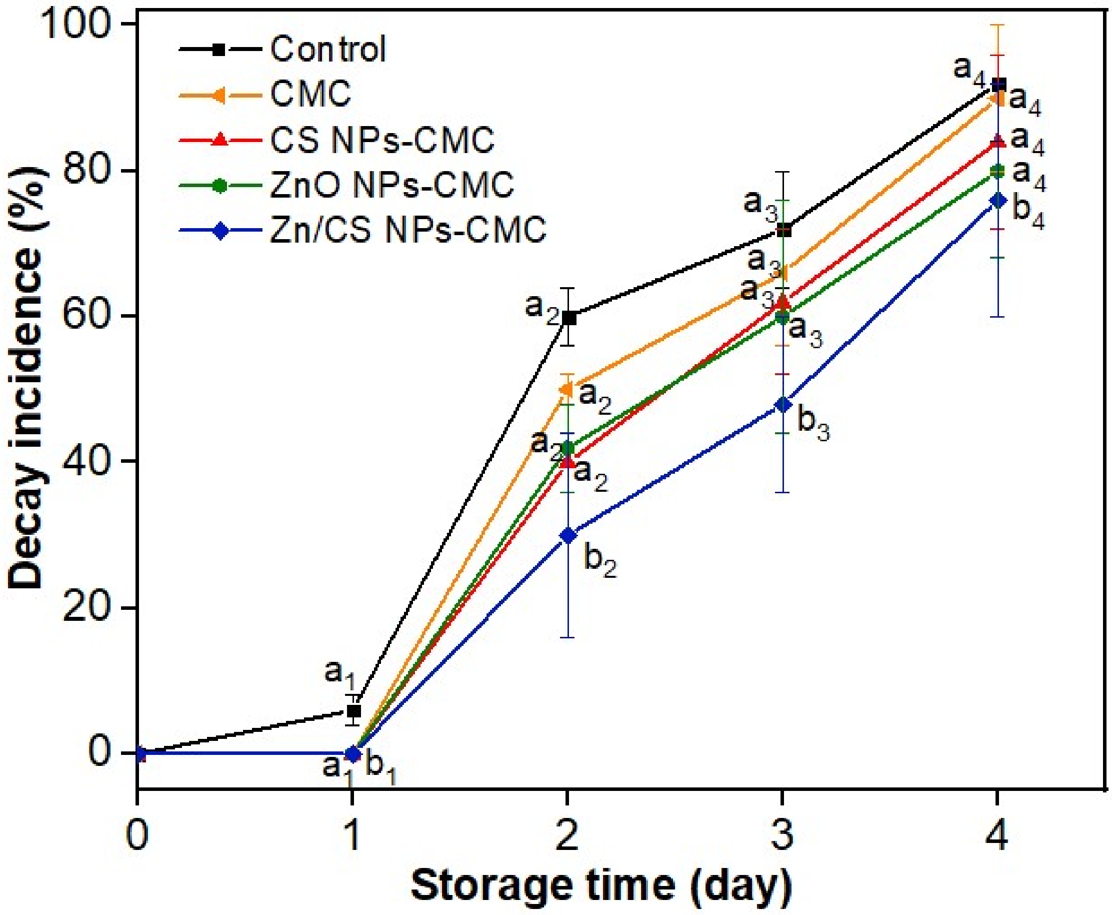

Supporting data 3. Decay incidence of control and coated strawberries stored at 25 °C
